# 16th International Conference on Human Retrovirology: HTLV and Related Retroviruses

**DOI:** 10.1186/1742-4690-11-S1-I1

**Published:** 2014-01-07

**Authors:** Benoit Barbeau

**Affiliations:** 1 Centre de recherche BioMed and Département des sciences biologiques, Université du Québec à Montréal, Montréal, Québec, Canada

## 

The 16th International Conference on Human Retrovirology: HTLV and Related Retroviruses took place in Montreal (Canada) from June 26^th^ to June 30^th^, 2013. The main topic covered during the meeting was human T-lymphotropic viruses (HTLVs) and was subdivided in distinct sessions: clinical research, animal models, immunology, molecular and cellular biology, human endogenous and emerging exogenous retroviruses and virology. The following abstracts were presented. Additional information about the conference can be found in Additional File 1.

## Supplementary Material

Additional File 1 Click here for file

