# Supplementary material for: 16th International Conference on Human Retrovirology: HTLV and Related Retroviruses
Source: Retrovirology. 2014 Jan 7;11(Suppl 1):I1. doi: 10.1186/1742-4690-11-S1-I1 (PMC4044609; doi:10.1186/1742-4690-11-S1-I1)
Supplement: Additional File 1 [file 1742-4690-11-S1-I1-S1.pdf]

SUPPLEMENTAL INFORMATION

Additional information about the supplement:

Acknowledgement

Gabriella Di Pancrazio (conference coordinator)
Mike Homme (Web site design)
AV Pro (audiovisual equipment and setting)
Christine Lalonde (graphics)
Natali Sakaryan and Johanne Galipeau (hotel liaison)

Canadian organizing committee members:

Benoit Barbeau (Université du Québec à Montréal)
John Hiscott (Vaccine & Gene Therapy Institute of Florida)
Greg Dekaban (John P. Robarts Institute)
Nikolaus Heveker (Université de Montréal)
Dixie Mager (University of British Columbia)
Joel Oger (University of British Columbia)
Éric Rassart (Université du Québec à Montréal)

Additional information about the conference:

http://www.htlv.net

Sponsorship:

BD Canada
Beckman Coulter LP
BioScience Writers
Canadian Institutes of Health Research (CIHR)
Cederlane
Centers for Retrovirus Research-The Ohio State University
Centre de recherche de l’Hôpital Ste-Justine
Illumina
Integrated DNA Technologies
Kyowa Hakko Kirin Co. Ltd.
Life Technologies
Rouge Maple
Sarstedt Canada
ThermoSolutions
Univalor
Université du Québec à Montréal (UQAM): Faculté des sciences and Vice-rectorat aux affaires publiques et aux relations gouvernementales et internationales
Université de Montréal: Faculté de médecine
Wisent Inc.
